# FGFR4 and EZH2 inhibitors synergistically induce hepatocellular carcinoma apoptosis via repressing YAP signaling

**DOI:** 10.1186/s13046-023-02659-4

**Published:** 2023-04-22

**Authors:** Yiqi Yang, Yibo Zhang, Jieqiong Cao, Zijian Su, Fu Li, Peiguang Zhang, Bihui Zhang, Rongzhan Liu, Linhao Zhang, Junye Xie, Jingsheng Li, Jinting Zhang, Xiaojia Chen, An Hong

**Affiliations:** 1 Institute of Biomedicine & Department of Cell Biology, College of Life Science and Technology, Guangdong Province Key Laboratory of Bioengineering Medicine, Guangdong Provincial Biotechnology Drug & Engineering Technology Research Center, National Engineering Research Center of Genetic Medicine, Ji’nan University, Guangzhou, 510632 China; 2 The First Affiliated Hospital, Ji’nan University, Guangzhou, 510630 China

**Keywords:** Hepatocellular carcinoma, Combination therapy, FGFR4, EZH2, Non-canonical NF-kB signaling, YAP signaling

## Abstract

**Background:**

Hepatocellular carcinoma (HCC) is one of the most common and lethal cancers worldwide, but current treatment options remain limited and cause serious life-threatening side effects. Aberrant FGFR4 signaling has been validated as an oncogenic driver of HCC, and EZH2, the catalytic subunit of the PRC2 complex, is a potential factor that contributes to acquired drug resistance in many tumors, including HCC. However, the functional relationship between these two carcinogenic factors, especially their significance for HCC treatment, remains unclear. In this study, we systematically evaluated the feasibility of a combination therapy targeting FGFR4 and EZH2 for HCC.

**Methods:**

RNA sequencing data of patients with Liver hepatocellular carcinoma (LIHC) from The Cancer Genome Atlas (TCGA) were analyzed to determine FGFR4 and EZH2 expression and their interaction with prognosis. Moreover, the HCC cell lines, zebrafish/mouse HCC xenografts and zebrafish HCC primary tumors were treated with FGFR4 inhibitor (Roblitinib) and/or EZH2 inhibitor (CPI-169) and then subjected to cell proliferation, viability, apoptosis, and tumor growth analyses to evaluate the feasibility of combination therapy for HCC both in vitro and in vivo. Furthermore, RNA-Seq was performed in combination with ChIP-Seq data analysis to investigate the critical mechanism underlying the combination treatment with Roblitinib and CPI-169.

**Results:**

EZH2 accumulated through the non-canonical NF-kB signaling in response to FGFR4 inhibitor treatment, and the elevated EZH2 levels led to the antagonism of HCC against Roblitinib (FGFR4 inhibitor). Notably, knockdown of EZH2 sensitized HCC cells to Roblitinib, while the combination treatment of Roblitinib and CPI-169 (EZH2 inhibitor) synergistically induced the HCC cell apoptosis in vitro and suppressed the zebrafish/mouse HCC xenografts and zebrafish HCC primary tumors development in vivo. Moreover, Roblitinib and CPI-169 synergistically inhibited HCC development via repressing YAP signaling.

**Conclusions:**

Collectively, our study highlighted the potential of the therapeutic combination of FGFR4 and EZH2 inhibitors, which would provide new references for the further development of clinical treatment strategies for HCC.

**Supplementary Information:**

The online version contains supplementary material available at 10.1186/s13046-023-02659-4.

## Background

Hepatocellular carcinoma (HCC) is the most common form of liver cancer, and its incidence is increasing worldwide [1]. Current chemotherapeutics, including sorafenib [2], lenvatinib [3] and regorafenib [4], provide limited efficacy for HCC patients and can cause significant side effects. Immunotherapy, such as the combination of atezolizumab and bevacizumab, might prolong the survival of HCC patients [5], but the long-term prognosis is still unclear. Poor diagnosis and limited treatment options result in a low survival rate for HCC patients. Thus, developing new strategies for the effective treatment of HCC remains an urgent priority.

Nearly 80% of HCC tumors harbor abnormal of fibroblast growth factors (FGFs) and/or their receptors (FGFRs) [6]. Among them, FGFR4 is highly expressed in liver tissue, and aberrant signaling of the FGF19-FGFR4 complex has been confirmed as a carcinogenic factor for HCC [7, 8]. In recent years, several small-molecule inhibitors targeting FGFR4 for the treatment of HCC and other solid tumors harboring aberrant FGFR4 signaling have entered clinical trials [7, 8]. However, the application of single-target drugs might have certain limitations, for they cannot entirely eliminate tumor cells and the long-term use of chemotherapy drugs might lead to drug resistance [7, 9]. Therefore, current mainstream research is focused on the suitability of cancers and whether these drugs can be combined with other drugs to enhance their efficacy.

Enhancer of zeste homology 2 (EZH2) is the catalytic subunit of the methyltransferase PRC2, which mediates histone 3 lysine 27 trimethylation (H3K27me3) and gene silencing [10, 11]. High levels of EZH2 in tissue from HCC patients are associated with tumor aggressiveness and poor prognosis [12], and previous studies suggested that EZH2 accumulation is a potential cause of acquired resistance to chemotherapy and immunotherapy [13–17]. Notably, we found that FGFR4 inhibitor treatment also resulted in EZH2 accumulation in HCC cells. Given this, we combined the FGFR4 inhibitor Roblitinib with the EZH2 inhibitor CPI-169 to observe the therapeutic effects on HCC.

In this study, we systematically investigated the feasibility of combination therapy targeting FGFR4 and EZH2 for HCC. We found that EZH2 expression was elevated by NFKB2 after FGFR4 inhibitor treatment, leading to the antagonism of HCC to FGFR4 inhibitors. Importantly, our results indicated that the combination treatment of Roblitinib (FGFR4 inhibitor) and CPI-169 (EZH2 inhibitor) synergistically induced HCC cell apoptosis in vitro and suppressed the zebrafish/mouse HCC xenografts and zebrafish HCC primary tumors development in vivo. Furthermore, we found that Roblitinib and CPI-169 synergistically inhibited HCC development by repressing YAP signaling. Overall, our data indicated that the combination of FGFR4 and EZH2 inhibitors can significantly inhibit tumor growth both in vitro and in vivo, which would supply new literatures for the development of clinical treatment strategies for HCC.

## Methods

### Cell culture, transfection and treatment

The human hepatoma cell lines HepG2, SMMC-7721, Huh7, MHCC97H, MHCC97L were cultured in high-glucose Dulbecco's modified Eagle medium (DMEM, Gibco) supplemented with 10% fetal bovine serum (FBS, Gibco) at 37 °C in a humidified atmosphere with 5% (v/v) CO2, while the human hepatic cell line THLE-2 was cultured under the same conditions in Dulbecco’s modified Eagles medium (DMEM)/F12 (Gibco) supplemented with 10% fetal bovine serum (FBS, Gibco). All cell lines were tested and confirmed free of mycoplasma.

Transfection were performed using the lipofectamine™ 3000 or RNAiMAX (Invitrogen) according to the manufacturer’s instruction. To inhibit FGFRs, cells were treated with the pan FGFR inhibitor Erdafitinib (1 uM) or the FGFR1/2/3 inhibitor AZD4547 (1 uM) for the indicated time before harvest. To inhibit FGFR4, cells were treated with the indicated concentrations of Roblitinib (Selleck), BLU9931 (Selleck) or H3B-6527 (Selleck) for the indicated time before harvest. To inhibit EZH2, cells were treated with the indicated concentrations of CPI-169 (Selleck) for the indicated time before harvest.

### Downloading of The Cancer Genome Atlas (TCGA) data and processing of RNA-seq data

A cohort of 419 human liver cancer specimens (including 369 LIHC and 50 paracancerous liver tissues) from The Cancer Genome Atlas (TCGA) database (https://www.cancer.gov/about-nci/organization/ccg/research/structural-genomics/tcga) was used to evaluate FGFR4 and EZH2 expression levels in liver cancer. Transcripts per million (TPM) was calculated and normalized using the Tag count comparison package (version 3.15; https://bioconductor.org/packages/release/bioc/html/TCC.html). The Kaplan–Meier method and log-rank test were used to evaluate the correlations between FGFR4 or EZH2 expression and OS patients with LIHC.

### Cell growth analysis

Cell proliferation was evaluated by Cell-Light™ EdU staining according to the manufacturer’s instructions. Cells were seeded at 2000 cells/well in 24-well plates and allowed to attach for 24 h prior to the indicated treatment (transfection or drug administration). The cells were finally dyed with Apollo (Red) and Hoechst 33,342 (Blue) and visualized under the fluorescence microscope. Cell viability was measured using Cell Counting Kit-8 assay according to the manufacturer’s instructions. Cells were seeded at 2000 cells/well in 96-well plates and allowed to attach for 24 h before the indicated treatment. Absorbance at OD450 was used to plot cell growth curves or to determine the half-maximal inhibitory concentration (IC50) of indicated drug in each cell lines. For the colony formation assay, cells were seeded (1000 cells/well) in 6-well plates for 24 h prior to the indicated treatment. The medium with the vehicle, Roblitinib and/or CPI-169 were replaced once a week and the positive wells were scored weekly as > 50% confluent.

### Drug interaction analysis

Cells were seeded at 2000 cells/well in 96-well plates and allowed to attach for 24 h, followed by treatment with increasing doses of Roblitinib and/or CPI-169 for 48 h. The cell viability was evaluated using Cell Counting Kit-8 assay and the drug interaction analysis was performed by Compusyn software (ComboSyn, Inc.) based on the combination index (CI) equation from Chou-Talalay method [18]. Drug interaction was scored as follows: CI = 1 is additive, CI < 1 is synergistic and CI > 1 is antagonistic.

### Zebrafish HCC xenografts injection and drug administration

The animal study was conducted according to the guidelines of the Declaration of Helsinki and approved by the Ethics Committee of Animal Experiments of Jinan University, Guangzhou, PR China. Zebrafish hepatocellular carcinoma xenografts injection were performed as described previously [19]. Briefly, mCherry-labeled HepG2 cells were injected into Subintestinal vein (SIV) of anesthetized 48-hpf larvae. After 24 h of injection, Zebrafish HCC xenografts with the same tumor sizes were randomly divided into 4 treatment groups: DMSO (1%), CPI-169 (40 µg/ml), Roblitinib (40 µg/ml) and combination therapy [CPI-169 (40 µg/ml) + Roblitinib (40 µg/ml)]. After 72 h treatment, the tumors in Zebrafish were photographed with a fluorescence microscope. The liver area and fluorescence intensity were measured by Image J software, and the tumor sizes were determined by the product of liver areas and fluorescence intensity.

### Generation of the Zebrafish KRAS^G12V+^, KRAS^G12V+^/EZH2^+^ and KRAS^G12V+^/YAP^S87A+^ HCC primary tumors and drug administration

Zebrafish KRAS^G12V+^ HCC primary tumors were generated as described previously [20, 21]. Briefly, the mutated (oncogenic) form of KRAS^G12V+^ fused to the mcherry sequence and subcloned into the pMDS6 vector driven by the liver-specifically expressed fabp10a promoter. Transgenic zebrafish were generated using the Ac/Ds transposon system as described previously [20, 21]. For drug administration, the anesthetized 48-hpf larvae were randomly divided into 4 groups, and treated with DMSO (1%), CPI-169 (40 µg/ml), Roblitinib (40 µg/ml) and combination therapy [CPI-169 (40 µg/ml) + Roblitinib (40 µg/ml)] for 72 h.

For the generation of the KRAS^G12V+^/EZH2^+^ HCC primary tumors, transgenic zebrafish containing the EZH2 gene driven by the fabp10a promoter were cross with the KRAS^G12V+^ transgenic zebrafish, and the transgenic zebrafish KRAS^G12V+^/EZH2^+^ were verified by genotyping using the indicated primers. For drug administration, the anesthetized 48-hpf larvae were randomly divided into 5 groups and treated with the indicated concentration of Roblitinib for 72 h.

For the generation of the KRAS^G12V+^/YAP1^S87A+^ HCC primary tumors, transgenic zebrafish containing the mutated (non-phosphorylated) form of YAP1^S87A+^driven by the fabp10a promoter were cross with the KRAS^G12V+^ transgenic zebrafish, and the transgenic zebrafish KRAS^G12V+^/YAP^S87A+^ were verified by genotyping using the indicated primers. For drug administration, the anesthetized 48-hpf larvae were treated with/without CPI-169 (40 µg/ml) + Roblitinib (40 µg/ml) for 72 h.

In all case, the tumors in Zebrafish were photographed with a fluorescence microscope. The liver area and fluorescence intensity were measured by Image J software, and the tumor sizes were determined by the product of liver areas and fluorescence intensity.

### Mouse HCC xenografts and drug administration

The animal study was conducted according to the guidelines of the Declaration of Helsinki and approved by the Ethics Committee of Animal Experiments of Jinan University, Guangzhou, PR China. BALB/c athymic nude mice (male, 4–5 weeks old) were purchased from the Experimental Laboratory Animal Center of Vital River (Beijing, China) and were housed in the animal facilities of Jinan University. Single-cell suspensions of 3 × 10^6^ SMMC-7721 cells were subcutaneously injected into the left flanks of the mice. When the tumors reached about 65 mm^3^, the mice were randomized into four groups (6 mice/group), and they were treated with vehicle, CPI-169 (30 mg/kg, oral administration), Roblitinib (20 mg/kg, oral administration), or a combination of both drugs. All drugs were administered every alternate day, and the tumor volume was measured at the indicated time points and calculated as length × width^2^/2.

### Immunohistochemistry (IHC)

Immunohistochemistry was performed as described previously [22]. Briefly, tumor tissues were paraffin-embedded and sectioned, followed by deparaffinization, rehydration and antigen retrieval. The tumor sections were then blocked with 3% BSA, followed by incubation with the indicated antibodies. The sections were then incubated with secondary antibodies and counterstained with hematoxylin, and then scanned with Axio Scan Z1 (Zeiss).

### Flow cytometry analysis of apoptosis

Cells (2 × 10^5^ cells/well) were seeded into 6-well plates for 24 h and the medium with the vehicle, Roblitinib and/or CPI-169 were added at the indicated concentrations for 48 h. For the cell apoptosis assay, cells were stained using the Annexin V-FITC Apoptosis Analysis Kit (BD Biosciences) and finally analyzed using the FACSAria™ flow cytometer (BD Biosciences).

### RNA extraction and Quantitative Real-time PCR (qPCR) assays

Total RNA was extracted and purified using TRIzol (Invitrogen) according to the manufacturer’s instructions, and 1 μg of total RNA was reverse transcribed using the PrimeScript RT Reagent Kit (TaKaRa). The gene expression levels were measured by a quantitative real-time PCR system (Bio-Rad). GAPDH was used as the reference gene for normalization. The qRT-PCR primers are listed in Table S1.

### Western blotting

Whole-cell extracts were obtained using RIPA buffer (50 mM Tris HCl (pH 8.0), 150 mM NaCl, 1% NP40, 0.5% sodium deoxycholate and 0.1% SDS) containing protease and phosphatase inhibitors Complete and PhosSTOP (Roche). The protein concentration of the lysate samples was determined by BCA protein assay kit (Pierce Biotechnology), and equal amounts of proteins (20 μg) were separated using 10% SDS–polyacrylamide gels and finally immunoblotted with the indicated antibodies. The information of the antibodies is listed in Table S2.

### RNA sequencing

Total RNA was isolated as described above, and approximately 1 μg RNA was used to construct RNA library with the NEBNext® Ultra™ Directional RNA Library Prep Kit for Illumina® (NEB). The raw paired-end reads were trimmed, and quality controlled by SeqPrep (https://github.com/jstjohn/SeqPrep) and Sickle (https:// github.com/najoshi/sickle) with default parameters. To identify differential expression genes (DEGs) between the two samples, the expression level of each transcript was calculated according to the fragments per kilobase of exon per million mapped reads (FPKM) method. Differential expression of genes (fold change ≥ 2 or ≤  − 2 and *p*-values < 0.05) were analyzed and selected for subsequent analysis.

### Statistical analysis

Statistical analysis was performed using the GraphPad Prism Software 9.0. The data were showed as mean ± standard error of the mean (SEM), and the statistical analyses were done using one-way or two-way ANOVA with Dunnett’s, Tukey’s or Sidak’s multiple comparison test (as indicated in the figure legends). Statistical differences with *p*-values < 0.05 were considered as significant. All experiments were repeated a minimum of three times.

## Results

### FGFR4 inhibitor treatment led to EZH2 accumulation by activating the non-canonical NF-kB signaling in HCC

**Fig. 1:**
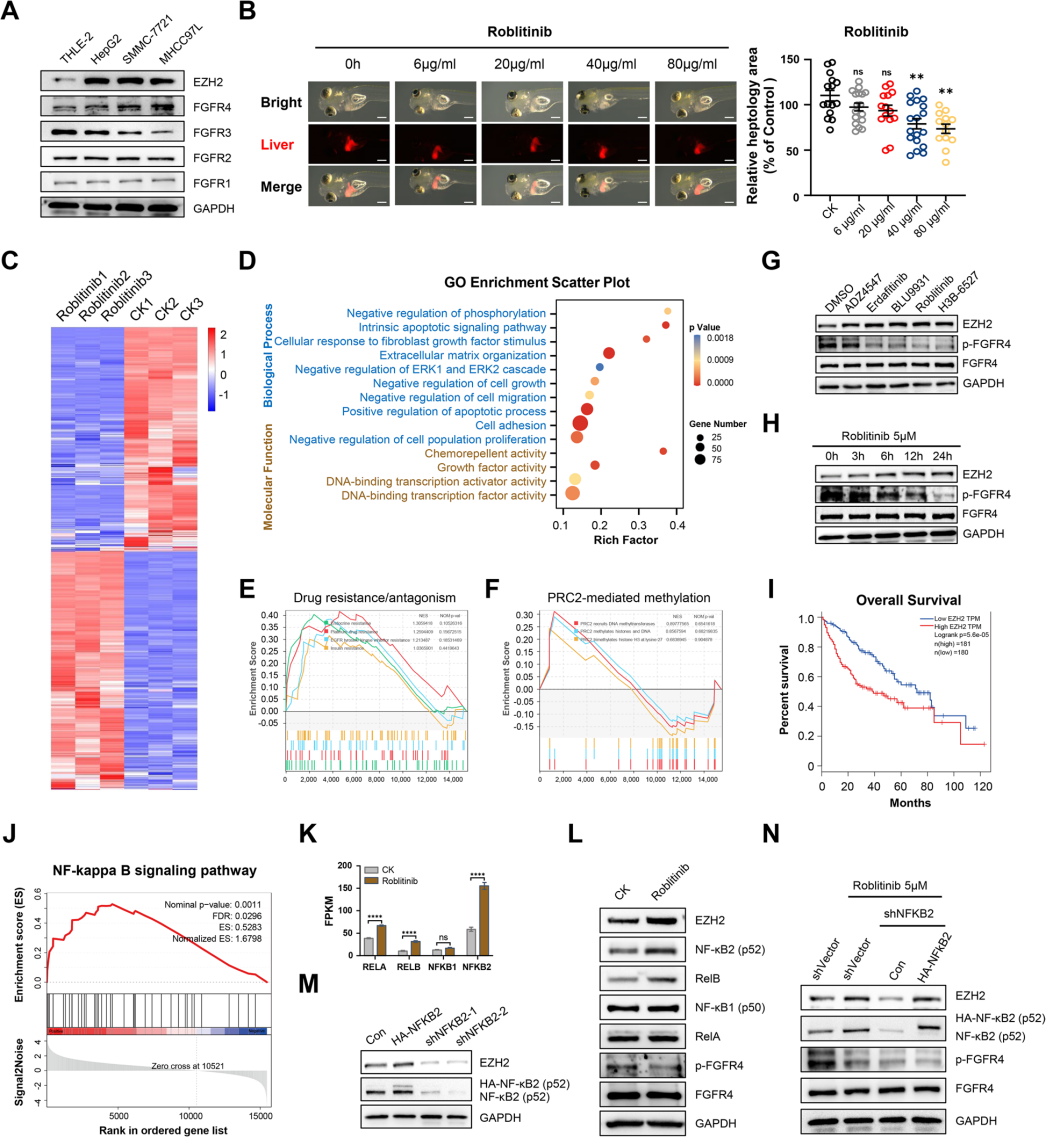
FGFR4 inhibitor treatment elevates EZH2 expression by activating non-canonical NF-kB signaling in HCC. **A** Western bolt detected the expression of EZH2 and FGFR1-4 in human hepatic cell line THLE-2 and human hepatoma cell lines HepG2, SMMC-7721 and MHCC97L. **B** Dose responses of zebrafish KRAS^G12V+^ HCC primary tumors treated with Roblitinib for 72 h. Scale bars: 100 μm. Data are presented as mean ± SEM (*n* = 15, one-way ANOVA with Dunnett’s multiple comparison test, **p < 0.01, ns, no significance). **C** Clustered heatmaps showed the differentially expressed genes between control group and Roblitinib treatment group (fold change ≥ 2 or ≤  − 2 and *p*-values < 0.05). HepG2 cells were treated with Roblitinib for 48 h, followed by RNA-Seq analysis. Red indicates high relative expression, and blue indicates low relative expression. **D** Bubble chart showed the Gene Ontology (GO) biological process and molecular function analysis of differentially expressed genes following Roblitinib treatment in (**C**). *P*-values < 0.05 was regarded as statistically significant. **E** Gene set enrichment analysis (GSEA) of the KEGG pathway showed that HCC cells treated with Roblitinib were enriched for the drug resistance/antagonism. **F** GSEA of Reactome pathway showed that cells treated with Roblitinib were enriched for transcriptional signatures associated with PRC2-mediated methylation. **G**-**H** Western bolt detected the expression of EZH2 after different FGFR inhibitor treatment for 48 h (**G**) and Roblitinib treatment for different durations (**H**). **I** High levels of EZH2 were associated with worse survival of HCC patients. **J** Gene set enrichment analysis (GSEA) showed that the NF-kB signaling pathway was enriched in the Roblitinib treatment group compared with Control group. **K** RNA-Seq analysis of the expression level of the indicated NF-kB genes after Roblitinib treatment for 48 h. The abscissa represented the indicated NF-kB genes, while the ordinate represented Fragments Per Kilobase of exon model per Million mapped fragments (FPKM). Data are presented as mean ± SEM (*n* = 3, two-way ANOVA with Sidak’s multiple comparison test, ****p < 0.0001, ns, no significance). **L** Western bolt detected the expression of the indicated NF-kB genes after Roblitinib treatment for 48 h. **M** Western blot analysis of EZH2 expression in HepG2 cells transfected with empty vector control (Con), HA-tagged NFKB2 (HA-NFKB2) and NFKB2-shRNAs (shNFKB2-1 and 2). **N** Western blot analysis of EZH2 expression in HepG2 cells after lentiviral transduction with control shRNA (shVector) or 3′ UTR NFKB2-targeting shRNA (shNFKB2) and restored with empty vector control (Con) or HA-tagged NFKB2 (HA-NFKB2), following by Roblitinib treatment for 48 h

Aberrant FGFR signaling has been reported in multiple tumor types [23, 24]. To confirm the key FGFRs in HCC development, we first examined their expression in human hepatic cells and several hepatoma cell lines, and we found that only FGFR4 was elevated in the representative HCC cell lines, whereas the other FGFRs were either unchanged or slightly downregulated (Fig. 1A). In addition, from the TCGA database, we found that FGFR4 expression was higher in LIHC tumor tissues than in para-cancer liver tissues, and higher FGFR4 expression was closely associated with poorer prognosis in HCC patients (Fig. S1A-C). Considering the functional importance of FGFR signaling in development, we used the FGFR4 inhibitors other than pan-FGFR or other FGFR inhibitors for HCC treatment.

Currently, several small-molecule inhibitors targeting FGFR4 have been investigated in clinical trial for the treatment of HCC [7, 8]. We consistently found that these FGFR4 inhibitors significantly inhibited the viability of multiple HCC cell lines (Fig. S2A-C). However, the application of Roblitinib in vivo might have a particular limitation since it could not fully inhibit cell growth in the zebrafish HCC primary tumors (Fig. 1B). To further confirm the critical mechanism mediating this response, RNA-Seq analysis was performed to compare gene expression profiles in HepG2 cells treated with or without Roblitinib (Fig. 1C). Gene Ontology (GO) biological process analysis showed that the differentially expressed genes were related to negative regulation of cell growth, migration and proliferation, but positive regulation of the apoptotic process (Fig. 1D). However, we noticed that a certain degree of drug resistance/antagonism in HCC cells treated with Roblitinib via the gene set enrichment analysis (GSEA) (Fig. 1E), which was consistent with what we observed in the zebrafish HCC primary tumors.

Previous studies have shown that aberrant histone methyltransferase is associated with drug resistance in multiply cancers, including HCC [25]. Interestingly, although unsignificant, we did identify several changes in transcriptional features associated with PRC2-mediated methylation in HCC cells following Roblitinib treatment via GSEA in the Reactome pathway (Fig. 1F), implying that PRC2-mediated methylation might play a role in the drug resistance/antagonism of HCC against Roblitinib. We then examined the expression level of EZH2, the catalytic subunit of PRC2, under the FGFR inhibitor treatment. Our results demonstrated that EZH2 expression increased after treatment with different FGFR inhibitors (Fig. 1G), and gradually accumulated with the duration of Roblitinib treatment (Fig. 1H). Importantly, higher EZH2 expression was strongly associated with higher cancer progression (Fig. S1D) and worse survival (Fig. 1I) in HCC patients.

To further confirm the critical factors mediating the accumulation of EZH2, we focused on the up-regulated genes induced by FGFR4 inhibitors. GSEA and KEGG pathway analysis identified that the NF-kB pathway was the core signaling pathway activated by Roblitinib treatment (Fig. 1J and S2D). Interestingly, we found that NFKB2, a transcription factor from the non-canonical NF-kB pathway that has been reported to directly induce EZH2 transcription [26], was up-regulated after the Roblinib treatment (Fig. 1K, L and S2E). To further explore the role of NFKB2 in EZH2 accumulation, we overexpressed or knocked down NFKB2 in HepG2 cells, and our results showed that EZH2 expression was correspondingly up- or down-regulated (Fig. 1M). Moreover, we knocked down NFKB2 in HepG2 cells followed by treatment with Roblitinib and found that the elevation in EZH2 was subsequently reversed, and its expression was restored once NFKB2 expression was rescued (Fig. 1N). These data indicated that the accumulation of EZH2 by FGFR4 inhibitor treatment relied on NFKB2.

Collectively, our data indicated that treatment with FGFR inhibitors, particularly FGFR4 inhibitors, may lead to EZH2 accumulation via activating non-canonical NF-kB signaling, whereas elevated EZH2 in HCC patients was associated with poor progression.

### Elevated EZH2 levels lead to antagonism of HCC against FGFR4 inhibitors

**Fig. 2:**
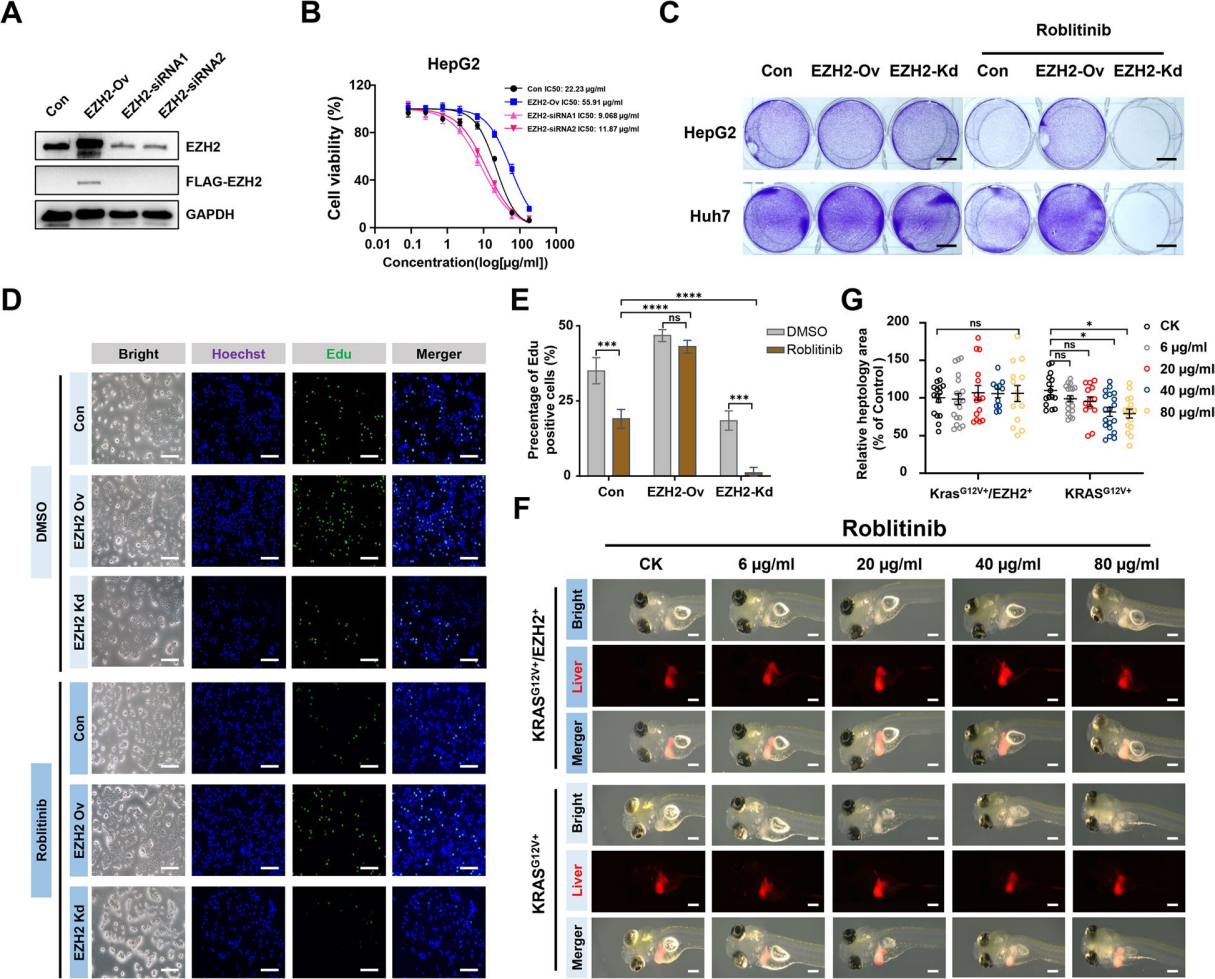
Elevated EZH2 levels lead to antagonism of HCC against FGFR4 inhibitors**.** **A** Western blot analysis of EZH2 expression in HepG2 cells transfected with empty vector control (Con), FLAG-EZH2 (EZH2-Ov) and EZH2-siRNAs (EZH2-siRNA1 and 2). **B** Cell viability of HepG2 cells transfected with empty vector control (Con), FLAG-EZH2 (EZH2-Ov) and EZH2-siRNAs (EZH2-siRNA1 and 2) was evaluated by the CCK-8 following increasing concentrations of Roblitinib treatment for 48 h. Data are presented as mean ± SEM (*n* = 3). **C** Crystal violet staining of HepG2 and Huh7 cell lines transfected with empty vector control (Con), FLAG-EZH2 (EZH2-Ov) and EZH2-siRNAs (EZH2-Kd) following Roblitinib treatment for 48 h. Scale bars: 1 cm. **D** EdU assays of HepG2 cells transfected with empty vector control (Con), FLAG-EZH2 (EZH2-Ov) and EZH2-siRNAs (EZH2-Kd) following Roblitinib treatment for 48 h. Scale bars: 100 μm. **E** Measurement of the cell numbers in (**D**). Data are presented as mean ± SEM (n = 3, two-way ANOVA with Sidak’s multiple comparison test, ***p < 0.001, ****p < 0.0001, ns, no significance). **F** Dose responses of zebrafish KRAS^G12V+^/EZH2^+^ (top) and KRAS^G12V+^ (bottom) HCC primary tumors treated with Roblitinib for 72 h. Scale bars: 100 μm. **G** Measurement of the tumor sizes in (**F)**. Data are presented as mean ± SEM (*n* = 15, two-way ANOVA with Sidak’s multiple comparison test, *p < 0.05, ns, no significance)

Previous studies have shown that EZH2 accumulation is a potential cause of acquired resistance to chemotherapy and immunotherapy [13–17]. To further elucidate the impact of EZH2 on HCC treatment, we ectopically expressed or knocked down EZH2 in HepG2 cells (Fig. 2A), followed by treatment with Roblitinib. Our results demonstrated that ectopic expression of EZH2 increased the viability (Fig. 2B, C, S3A-C) and proliferation (Fig. 2D, E, S3D and E) of HCC cells after Roblitinib treatment, leading to the antagonism, while under the same conditions, knockdown of EZH2 reduced cell viability (Fig. 2B, C, S3A-C) and proliferation (Fig. 2D, E, S3D and E), suggesting that knockdown of EZH2 may sensitize HCC cells to Roblitinib. To further investigate the influence of EZH2 on FGFR4 inhibitor treatment in vivo, we generated zebrafish HCC primary tumors with liver-specific overexpression of EZH2 (KRAS^G12V+^/EZH2^+^). Consistently, the tumor sizes in the zebrafish HCC primary tumors (KRAS^G12V+^) decreased with Roblitinib treatment, while in the zebrafish HCC primary tumors containing ectopic EZH2 expression, Roblitinib failed to inhibit the HCC cell growth (Fig. 2F and G). Therefore, increased EZH2 levels were responsible for the antagonism of HCC against FGFR4 inhibitor treatment.

### Combination of Roblitinib and CPI-169 synergistically inhibited HCC development

**Fig. 3:**
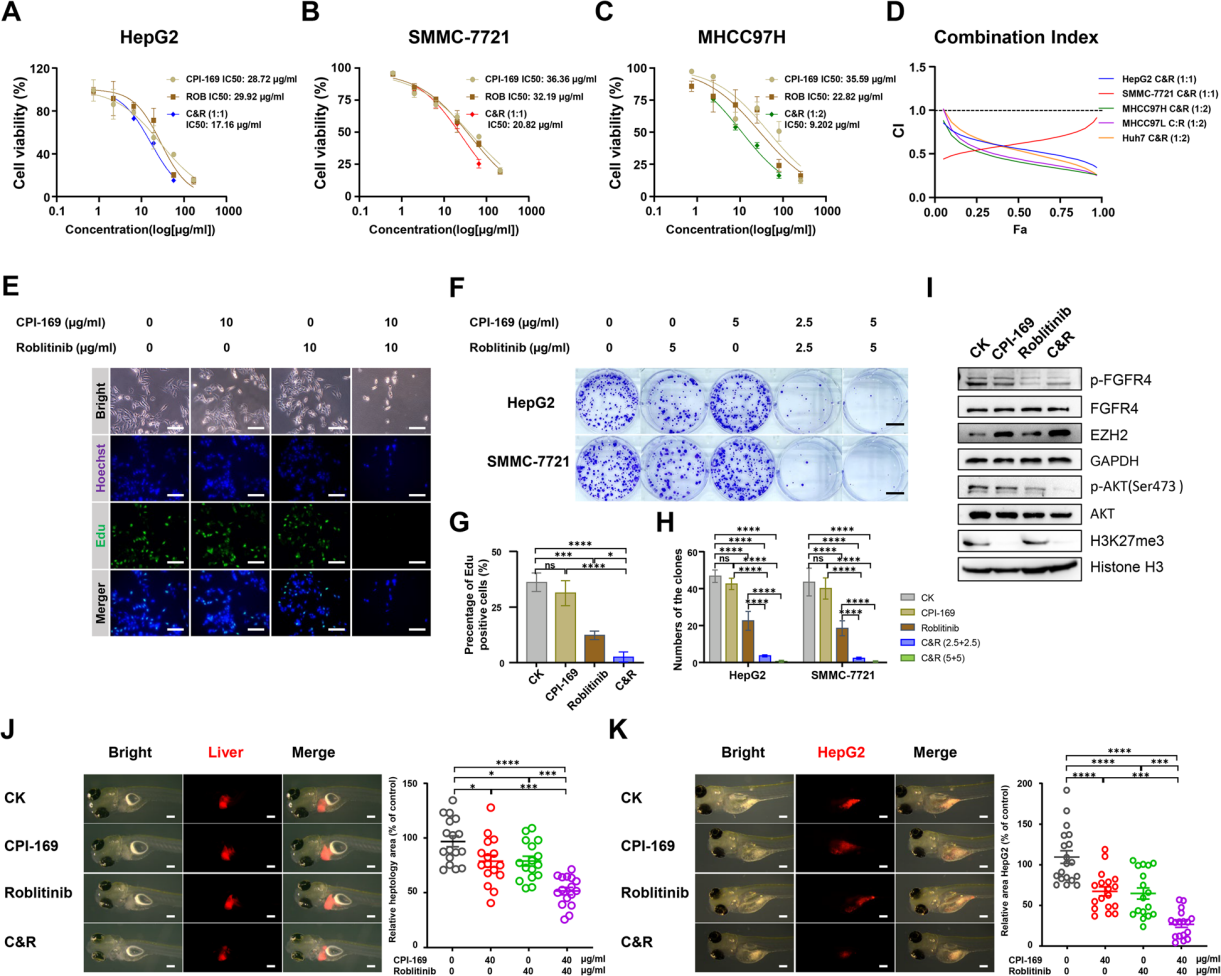
Combination of Roblitinib and CPI-169 synergistically inhibits the HCC cell growth. **A**-**C** Cell viability of HepG2 (**A)**, SMMC-7721 (**B**) and MHCC97H (**C**) cell lines was evaluated by the CCK-8 following increasing concentrations of CPI-169, Roblitinib or CPI-169 + Roblitinib treatment for 48 h. Data are presented as mean ± SEM (*n* = 3). **D** Drug interaction analysis between CPI-169 and Roblitinib in HepG2, SMMC-7721, MHCC97H, MHCC97L and Huh7 cell lines. The CI values less than 1.0, approximately 1.0, and greater than 1.0 indicate synergism, additive, and antagonism, respectively. **E** EdU assay of HepG2 cells following CPI-169, Roblitinib or CPI-169 + Roblitinib treatment for 48 h. Scale bars: 50 μm. **F** Colony formation assay of HepG2 and SMMC-7721 cell lines following CPI-169, Roblitinib or CPI-169 + Roblitinib treatment for 14 days. Scale bars: 1 cm. **G** Measurement of the cell numbers in (**E**). Data are presented as mean ± SEM (*n* = 3, one-way ANOVA with Tukey’s multiple comparison test, *p < 0.05, ***p < 0.001, ****p < 0.0001, ns, no significance). **H** Measurement of the clone numbers in (**F**). Data are presented as mean ± SEM (*n* = 3, two-way ANOVA with Sidak’s multiple comparison test, ****p < 0.0001, ns, no significance). **I** Western blot analysis of the indicated protein expression in HepG2 cells following CPI-169, Roblitinib or CPI-169 + Roblitinib treatment for 48 h. **J**-**K** Zebrafish harboring KRAS^G12V+^ HCC primary tumors (**J**) and zebrafish HCC xenografts using the mCherry-labbled HepG2 cells (K) treated with CPI-169, Roblitinib or CPI-169 + Roblitinib for 72 h. Scale bars: 100 μm. Data are presented as mean ± SEM (*n* = 15, one-way ANOVA with Tukey’s multiple comparison test, *p < 0.05, ***p < 0.001, ****p < 0.0001, ns, no significance)

Given that EZH2 knockdown sensitized HCC cells to FGFR4 inhibitors, we then investigated their synergistic effect by simultaneously inhibiting FGFR4 and EZH2. CCK8, EdU, and colony formation assays were performed in several HCC cell lines following treatment with Roblitinib and/or CPI-169, and our results demonstrated that the combination treatment exhibited more potent effects on reducing cell viability (Fig. 3A-D, F, H, S4A-L) and cell proliferation (Fig. 3E and G) than the single inhibitor treatment alone. Moreover, the combination index (CI) was calculated, and the results showed convincing synergism of the combined use of Roblitinib and CPI-169 (CI < 1.0) in several HCC cell lines (Fig. 3D, S4F-J). Consistently, double knockdown of FGFR4 and EZH2 exerted stronger inhibitory effects on HCC cell proliferation than single knockdown of FGFR4 or EZH2 (Fig. S4M). These results indicated that the combined inhibition of FGFR4 and EZH2 synergistically inhibited cell viability and proliferation in vitro.

To investigate whether FGFR4 and EZH2 inhibitors could synergistically repress tumor growth in vivo, we first administered Roblitinib and CPI-169 alone or in combination in the zebrafish HCC primary tumors. As shown in Fig. 3J, both Roblitinib and CPI-169 inhibited the growth of primary tumors individually, while the combination treatment showed more obvious inhibitory effects. Consistent results were also found in our zebrafish HCC xenografts using mCherry-labeled HepG2 cells, where the combination of Roblitinib and CPI-169 showed stronger inhibition than either treatment alone (Fig. 3K).

**Fig. 4:**
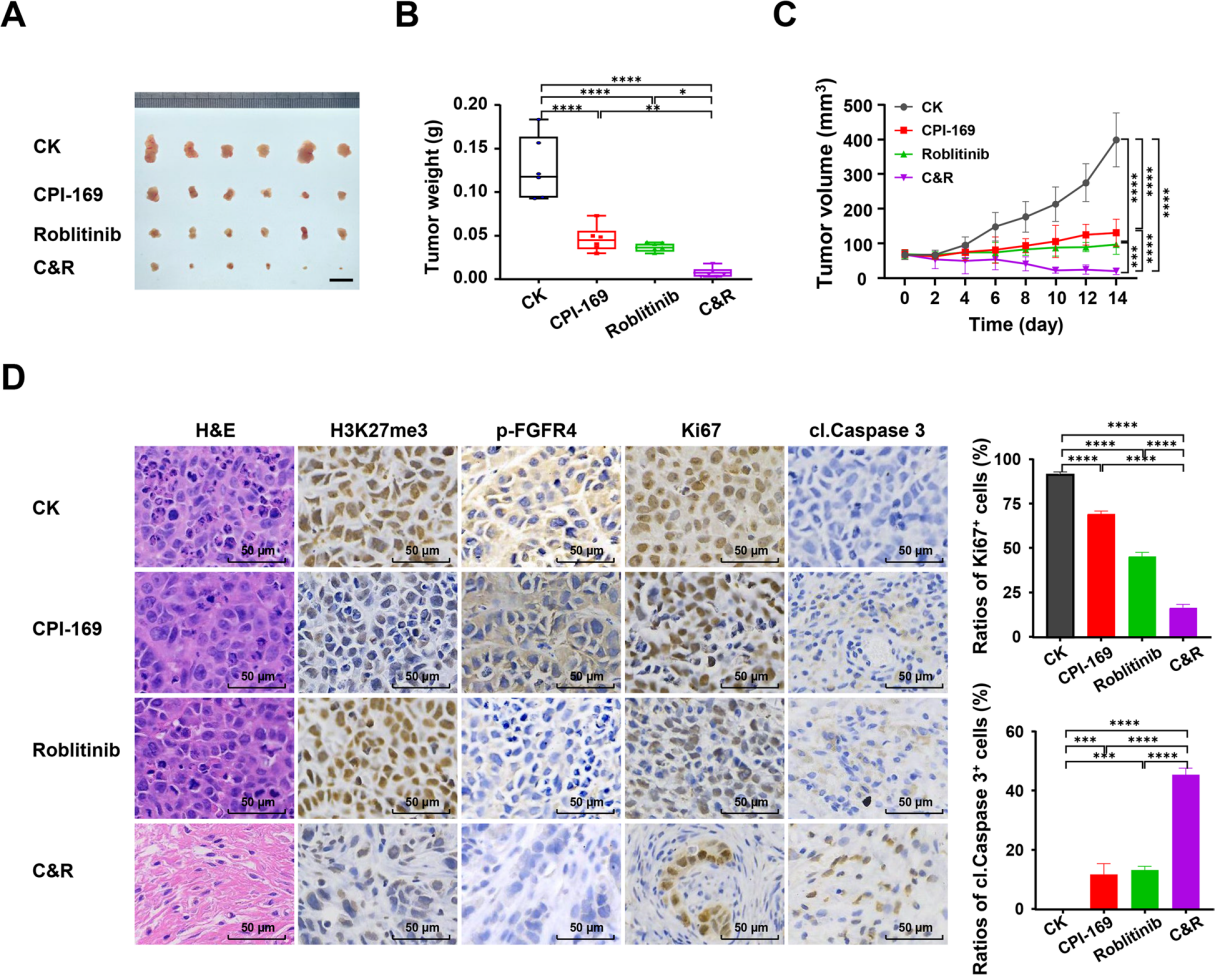
Combination of Roblitinib and CPI-169 elicits robust anti-cancer effects in vivo. **A**-**C** Subcutaneous xenograft tumors established from SMMC-7721 cells were treated with vehicle or the indicated drugs. Shown are the tumors (**A**), tumor weight (**B**) and the tumor volume (**C**) from the recipient mice. Mice with SMMC-7721 xenografts were treated with vehicle (*n* = 6), CPI-169 (*n* = 6), Roblitinib (*n* = 6) or CPI-169 + Roblitinib (*n* = 6) for 2 weeks. Scale bars: 1 cm. Values are presented as mean ± SEM; One-way ANOVA with Tukey’s multiple comparisons test, *p < 0.05, **p < 0.01, ***p < 0.001, ****p < 0.0001. **D** Representative H&E and IHC staining for H3K27me3, phospho-FGFR4 (p-FGFR4), Ki67 and Cleaved caspase-3 (cl. Caspase 3) in tumors. Scale bars: 50 μm. The intensity of Ki67 and Cleaved caspase-3 staining cells were quantified. Values are presented mean ± SEM; One-way ANOVA with Tukey’s multiple comparisons test, ***p < 0.001, ****p < 0.0001

To further confirm the combined effect of Roblitinib and CPI-169 in vivo, SMMC-7721 cells were inoculated into the BALB/C nude mice. Consistent with the in vitro and zebrafish results, treatment with either Roblitinib or CPI-169 decreased tumor volumes, and their combination further prevented tumor growth (Fig. 4A-C). Moreover, immunohistochemistry (IHC) results showed decreased Ki67 expression but increased cleaved caspase-3 expression in tumors treated with the combination of Roblitinib and CPI-169 compared with the untreated or single agent-treated tumors (Fig. 4D), suggesting that combination treatment elicited a robust anti-cancer effect in vivo. Notably, the survival rate, body length and heartbeat of zebrafish did not change significantly within 72 h after treatment with CPI-169 and/or Roblinib (Fig. S5), and the weight, heart, liver, spleen, lung and kidney of representative mice treated with CPI-169 and/or Roblitinib for 2 weeks also did not show obvious changes (Fig. S6), indicating that Roblitinib, CPI-169 and their combination were well tolerated in zebrafish and mice with no observed toxic effects.

Taken together, our results demonstrated that the combination of Roblitinib and CPI-169 synergistically inhibited HCC development both in vitro and in vivo.

### Combination of Roblitinib and CPI-169 synergistically induced HCC cell apoptosis

Previous studies have shown that FGFR4 and EZH2 inhibitors can induce cell apoptosis in various cancer cells [7, 11]. Hence, to elucidate whether the combination of Roblitinib and CPI-169 could enhance cell apoptosis, HCC cell lines were cultured in vitro, followed by treatment with Roblitinib and/or CPI-169. Our results showed that treatment with Roblitinib or CPI-169 alone had a slight effect on HCC cell viability, while their combination significantly reduced cell viability and induced mass cell death (Fig. S7A). We then investigated the influence of the combination treatment on cell apoptosis by flow cytometry analysis. Apoptotic cell numbers were quantified by Annexin V-fluorescein isothiocyanate (FITC) and propidium iodide (PI) double staining, and the results showed that the percentage of early and late apoptotic cells in the combined treatment group was increased significantly compared with the single treatment groups (Fig. S7B). We finally examined the effect of the combination treatment on apoptosis-related protein expression, and our results showed that compared with the Roblitinib or CPI-169 group, the expression of PARP and caspase-3 was decreased in the combination group (Fig. S7C). In contrast, the levels of cleaved Caspase-3 and cleaved PARP were increased (Fig. S7C), and similar results were found in our mouse HCC xenografts, where the combination of Roblitinib and CPI-169 induced more cleaved Caspase-3 than either treatment alone (Fig. 4D). Together, these data indicated that Roblitinib and CPI-169 synergistically induced HCC cell apoptosis.

### Combination of Roblitinib and CPI-169 synergistically inhibited the YAP signaling

**Fig. 5:**
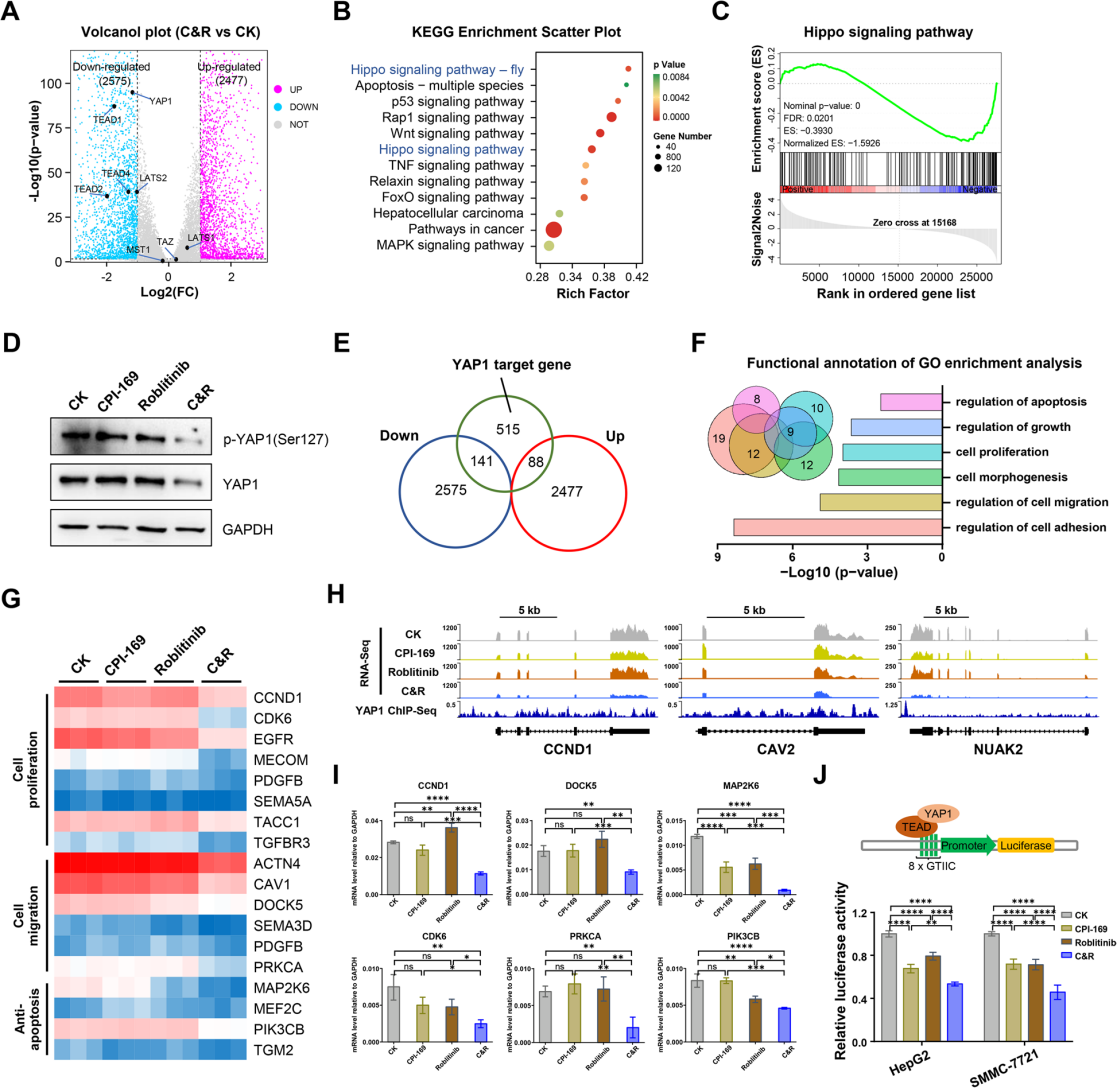
Combination of Roblitinib and CPI-169 synergistically inhibits the YAP signaling**.** **A** Volcano Plot showed the differentially expressed genes between control group and CPI-169 + Roblitinib treatment group (fold change ≥ 2 or ≤  − 2 and *p*-values < 0.05). HepG2 cells were treated with CPI-169 + Roblitinib for 48 h, followed by RNA-Seq analysis. Red indicates high relative expression, and blue indicates low relative expression. **B** Bubble chart showed the KEGG pathway analysis of differentially expressed genes following CPI-169 + Roblitinib treatment in (**A)**. *P*-values < 0.05 was regarded as statistically significant. **C** Gene set enrichment analysis (GSEA) showed that the Hippo signaling pathway was enriched in the co-treatment group compared with Control group. **D** Western bolt detected the expression level of YAP1 and p-YAP1 after CPI-169, Roblitinib or CPI-169 + Roblitinib treatment for 48 h. **E** Venn diagram showed YAP1 target genes that were regulated by CPI-169 + Roblitinib treatment. **F** Gene functional annotation of GO enrichment analysis of the 141 down-regulated YAP1 target genes in (**E**) via Metascape. **G** Heatmap representing the expression levels of 18 YAP1 target genes related to cell proliferation, cell migration and anti-apoptosis determined by RNA-Seq in HepG2 cells following CPI-169, Roblitinib or CPI-169 + Roblitinib treatment for 48 h. **H** Genomic tracks displayed ChIP-seq data for YAP1 around the indicated genes and their corresponding RNA-Seq signals in (**G**). **I** qPCR analysis of the representative YAP1 target genes from typical pathway related to cell proliferation, cell migration and anti-apoptosis in (**G**). Data are presented as mean ± SEM (*n* = 3, one-way ANOVA with Tukey’s multiple comparisons test, *P < 0.05, **P < 0.01, ***p < 0.001, ****p < 0.0001, ns, no significance). **J** Luciferase assay for YAP/TAZ activity in HepG2 and SMMC-7721 cells following CPI-169, Roblitinib or CPI-169 + Roblitinib treatment for 48 h. Data are presented as mean ± SEM (*n* = 3, two-way ANOVA with Sidak’s multiple comparison test, **P < 0.01, ****p < 0.0001)

Our results presented so far indicated that the combination of Roblitinib and CPI-169 can synergistically induce HCC cell apoptosis, thereby inhibiting HCC development. To elucidate the critical mechanism in response to the combination treatment, we performed RNA sequencing analysis comparing gene expression profiles in HepG2 cells treated with or without Roblitinib and/or CPI-169 (Fig. S8A). Simultaneous inhibition of both FGFR4 and EZH2 resulted in the most dramatic changes in the HepG2 cells transcriptome compared to the control group (Fig. 5A and S8B), with 6255 differentially expressed genes, which was far more than that of the CPI-169 or Roblitinib treatment groups, with a total of 643 and 2332 differentially expressed genes (Fig. S8B), respectively. KEGG pathway analysis of the differentially expressed genes identified the Hippo signaling pathway as a critical pathway affected by combination treatment (Fig. 5B), and the GSEA also showed that the gene sets related to the Hippo signaling pathway were significantly downregulated (Fig. 5C), suggesting that combination treatment with Roblitinib and CPI-169 synergistically regulates the Hippo signaling pathway.

Aberrant Hippo signaling has been reported to be implicated in cancer cell growth, proliferation and migration [27, 28]. Notably, we found that the combination of Roblitinib and CPI-169 synergistically inhibited YAP1 gene expression compared to the single treatments (Fig. 5A, D and S8D). The activity of YAP protein is negatively regulated by the kinase LATS1/2 [28]; however, we noticed that both the expression and phosphorylation level of the upstream MST1/2 and LAST1/2 were not obviously changed in the combination treatment group (Fig. 5A and Fig. S8C), suggesting that Roblitinib and CPI-169 was likely to synergistically interfere with the downstream Hippo signaling by repressing YAP expression.

To further confirm whether YAP signaling is involved in this response, we reanalyzed YAP1 ChIP-seq data from human cholangiocarcinoma cell lines and identified 741 YAP1-binding genes [29] (Fig. S8E). These identified binding genes were cross-referenced with the down-regulated genes induced by combination treatment, and thus, 141 direct and conserved YAP transcriptional targets were selected (Fig. 5E). The expression of these 141 genes decreased slightly after treatment with Roblitinib or CPI-169 alone, but significantly after treatment with both in combination (Fig. S8F), and the gene functional annotation of GO enrichment analysis via Metascape indicated that 40 of these 141 genes were related to cell proliferation (such as CCND1 and CDK6), migration (such as DOCK5 and PRKCA) and anti-apoptosis (such as MAP2K6 and PIK3CB) pathways (Fig. 5F-I), suggesting that the combination of Roblitinib and CPI-169 may repress several oncogenic gene expression programs by inhibiting YAP. Finally, a YAP/TAZ luciferase reporter was transfected into the HepG2 and SMMC-772 cell lines, followed by treatment with Roblitinib and/or CPI-169, and consistently, simultaneous inhibition of FGFR4 and EZH2 resulted in the most dramatic changes in luciferase activity (Fig. 5J).

Collectively, our results indicated that the combination of Roblitinib and CPI-169 synergistically inhibited YAP signaling and its downstream cell proliferation, migration and anti-apoptotic programs.

### Overexpression of YAP1^S127A^ antagonized the synergistic inhibitory effect of Roblitinib and CPI-169 on HCC development

**Fig. 6:**
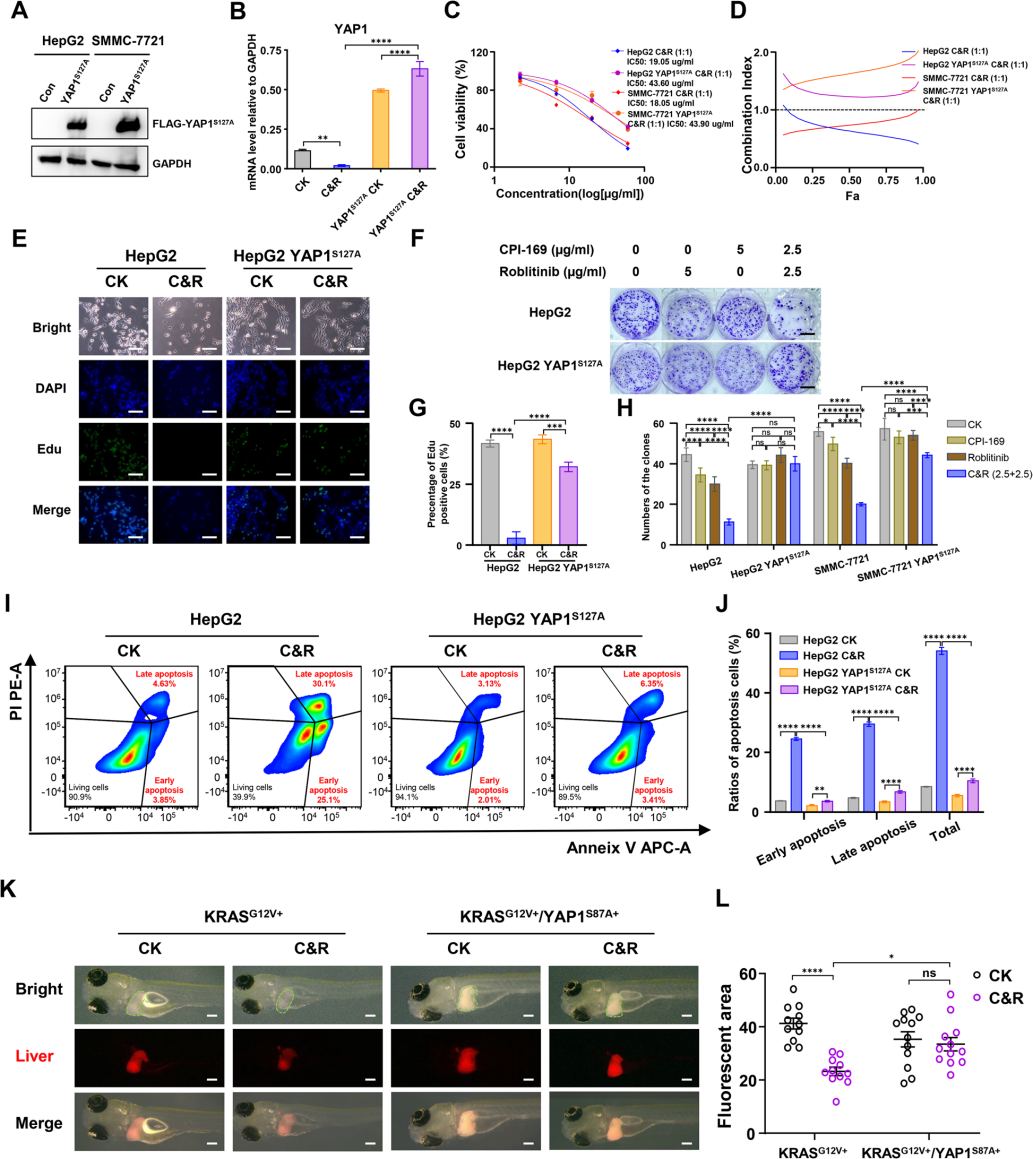
Overexpression of YAP1^S127A^ antagonizes the synergistic inhibitory effect of Roblitinib and CPI-169 in HCC cells. **A** Western blot analysis of FLAG-YAP1^S127A^ expression in HepG2 and SMMC-7721 cell lines stably transfected with empty vector control (Con) or FLAG-YAP1^S127A^ (YAP1^S127A^). **B** qPCR analysis of YAP1 mRNA level in HepG2 and HepG2 YAP1^S127A^ cell lines following the CPI-169 + Roblitinib treatment for 48 h. Data are presented as mean ± SEM (*n* = 3, two-way ANOVA with Sidak’s multiple comparison test, ****p < 0.0001, ns, no significance). **C** Cell viability of HepG2, HepG2 YAP1^S127A^, SMMC-7721 and SMMC-7721 YAP1^S127A^ cell lines was evaluated by the CCK-8 following increasing concentrations of CPI-169 + Roblitinib treatment for 48 h. Data are presented as mean ± SEM (*n* = 3). **D** Drug interaction analysis between CPI-169 and Roblitinib in HepG2, HepG2 YAP1^S127A^, SMMC-7721 and SMMC-7721 YAP1^S127A^ cell lines. The CI values less than 1.0, approximately 1.0 and greater than 1.0 indicate synergism, additive and antagonism, respectively. **E** EdU assays of HepG2 and HepG2 YAP1^S127A^ cell lines following the CPI-169 + Roblitinib treatment for 48 h. Scale bars: 50 μm. **F** Colony formation assay of HepG2 and HepG2 YAP1^S127A^ cell lines following CPI-169, Roblitinib or CPI-169 + Roblitinib treatment for 14 days. Scale bars: 1 cm. **G** Measurement of the cell numbers in (**E**). Data are presented as mean ± SEM (*n* = 3, two-way ANOVA with Sidak’s multiple comparison test, ***p < 0.001, ****p < 0.0001). **H** Measurement of the clone numbers in (**F**) and SMMC-7721 and SMMC-7721 YAP1^S127A^ cell lines following CPI-169 and/or Roblitinib treatment for 14 days. Data are presented as mean ± SEM (*n* = 3, two-way ANOVA with Sidak’s multiple comparison test, *p < 0.05, ***p < 0.001, ****p < 0.0001, ns, no significance). **I**-**J** Flow cytometric analysis of apoptosis in HepG2 and HepG2 YAP1^S127A^ cell lines following CPI-169 + Roblitinib treatment for 48 h. Data are presented as mean ± SEM (*n* = 3, two-way ANOVA with Sidak’s multiple comparison test, **p < 0.01, ****p < 0.0001). **K**-**L** Zebrafish harboring KRAS^G12V+^ (left) or KRAS^G12V+^/YAP1^S87A+^ (right) HCC primary tumors were treated with CPI-169 + Roblitinib for 72 h. Scale bars: 100 μm. Data are presented as mean ± SEM (*n* = 12, two-way ANOVA with Sidak’s multiple comparison test, *p < 0.05, ****p < 0.0001 ns, no significance)

To investigate whether Roblitinib and CPI-169 synergistically inhibit HCC development in a YAP signaling-dependent manner, a YAP1 mutant construct YAP1^S127A^, which stimulates YAP1 binding to TEAD1, was stably transfected into HepG2 and SMMC-7721 cell lines (Fig. 6A). Notably, combination treatment with Roblitinib and CPI-169 significantly inhibited YAP1 expression in HCC cell lines but had little effect on cells expressing YAP1^S127A^ (Fig. 6B). We then performed CCK-8 (Fig. 6C, S9A and B), EdU (Fig. 6E and G), colony formation (Fig. 6F, H and S9C) and flow cytometry analysis (Fig. 6I and J) in HCC cell lines and those expressing the YAP1^S127A^ gene following combination treatment. Our results showed that the combination treatment of Roblitinib and CPI-169 significantly inhibited cell viability, proliferation and induced apoptosis in HCC cell lines, while these effects were attenuated in HCC cells expressing YAP1^S127A^ (Fig. 6C, E-J, S9A-C). Moreover, the combination index (CI) was calculated, and the results showed that the combination treatment exhibited convincing synergistic effects on HCC cell lines, but these effects were antagonized in cells expressing YAP1^S127A^ (Fig. 6D). These data suggest that overexpression of YAP1^S127A^ antagonized the synergistic inhibitory effect of Roblitinib and CPI-169 on HCC cell growth.

To further confirm that Roblitinib and CPI-169 synergistically induce HCC cell apoptosis depends on YAP signaling, we first took advantage of a luciferase reporter gene system to examine the YAP/TAZ activity of HepG2 cells, SMMC-7721 cells and cells expressing YAP1^S127A^ following the combination treatment. Our results indicated that combination treatment significantly inhibited YAP/TAZ activity in HCC cell lines but had little effect on those expressing YAP1^S127A^ (Fig. S9D). We then measured the expression level of a series of YAP direct target genes related to cell proliferation, migration and anti-apoptotic pathways in HepG2 and HepG2YAP1^S127A^ cell lines after the combination treatment. Our results indicated that the combination treatment significantly inhibited the YAP target gene expression in HCC cell lines but had little effect on cells expressing YAP1^S127A^ (Fig. S9E), suggesting that combination treatment with Roblitinib and CPI-169 repressed the oncogenic gene expression programs, including the anti-apoptotic gene expression programs, in a YAP signaling-dependent manner.

To further investigate the influence of YAP on FGFR4 and EZH2 inhibitor treatment in vivo, we generated zebrafish HCC primary tumors with liver-specific overexpression of YAP1^S87A^ (KRAS^G12V+^/YAP1^S87A+^). Consistent with these findings, the tumor sizes in zebrafish HCC primary tumors decreased with the combination treatment of the Roblitinib and CPI-169, whereas the tumors containing ectopic YAP1^S87A+^ expression showed remarkable antagonism to the treatment (Fig. 6K and L). These data indicated that combination of Roblitinib and CPI-169 synergistically inhibited zebrafish HCC primary tumors growth via repressing YAP signaling in vivo.

Together, our results indicated that the combination of Roblitinib and CPI-169 synergistically inhibited HCC development in a YAP signaling-dependent manner.

## Discussion

**Fig. 7:**
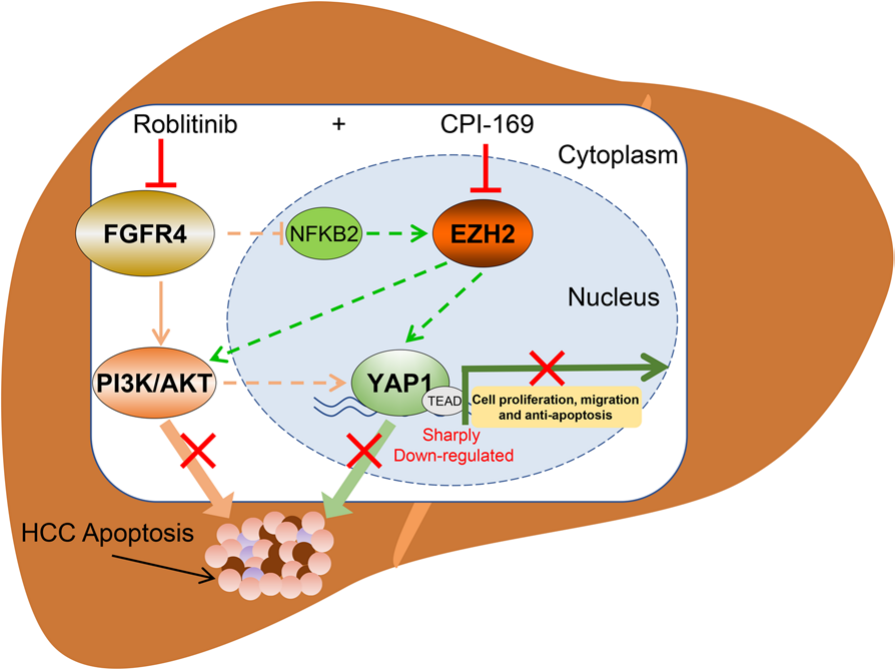
Proposed model for synergetic induction of apoptosis in HCC by FGFR4 and EZH2 inhibitors. Treatment with FGFR4 inhibitors alone induced EZH2 accumulation by activating NF-kB2, which maintained YAP expression levels to maintain some degree of cell proliferation-, migration- and anti-apoptosis-related genes expression, resulting in antagonism to Roblitinib. Only when FGFR4 and EZH2 were simultaneously suppressed would YAP expression and its corresponding signaling decrease sharply and eventually synergistically induce substantial HCC cell apoptosis

Although diagnostic tools and therapies have been designed, currently available treatment options for HCC are still limited, and thus, developing effective strategies to combat this deadly disease remains urgent. In the present study, we determined that Roblitinib, a representative FGFR4 inhibitor, exerted anti-cancer effects in HCC cells, and these effects can be enhanced by EZH2 inhibitor CPI-169. Furthermore, we found that inhibition of both FGFR4 and EZH2 showed a synergistic effect on HCC treatment via repressing YAP signaling, which might provide a potential option for HCC patients in the future (Fig. 7).

Epigenetic regulation plays vital roles in cell growth, differentiation and apoptosis, and its dysregulation is closely related to tumorigenesis and progression, and in addition, aberrant histone modifications are often accompanied by cancer drug resistance [25]. In the present study, we found that the administration of FGFR4 inhibitors, on one hand, can significantly inhibit the HCC cell growth via suppressing the downstream PI3K/AKT pathways (Fig. 3I), and on the other hand, may result in the accumulation of EZH2 by activating the non-canonical NF-kB signaling transcription factor NFKB2 (Fig. 1J-N), leading to the failure of Roblitinib to eliminate tumor cells (Fig. 1B and 2B-F). Moreover, EZH2 has been reported to activate PI3K/AKT pathway and induce acquired resistance in NSCLC [14], and consistent with these findings, we found that inhibition of the EZH2 led to a slight downregulation of AKT and p-AKT levels (Fig. 3I). Thus, elevated EZH2 levels caused by FGFR4 inhibitors might also regulate the PI3K/AKT pathway, leading to the antagonism to Roblitinib in HCC. Previous studies have shown that concomitant use of EZH2 inhibitors can prevent the development of acquired resistance, thereby enhancing the efficacy of chemotherapy and immunotherapy [16, 30–32]. Based on this, we combined Roblitinib and CPI-169 in the treatment of HCC and found that they exerted a synergistic effect on inducing HCC cell apoptosis (Fig. 4 and S7).

Strikingly, we found that co-administration of FGFR4 and EZH2 inhibitors synergistically repressed YAP signaling to induce HCC cell apoptosis, rather than regulating the upstream MST1/2 and LAST1/2 signaling (Fig. 5B-I and S8C). The Hippo signaling pathway plays critical roles in development, homeostasis, and tumor progression [28, 33]. As an oncogene and transcriptional co-factor, YAP targets many cell cycle-, migration-, and anti-apoptosis-related genes to regulate the HCC development [28, 33]. Previous studies have shown that FGFR4 signaling inhibits the activity of YAP by phosphorylating the upstream MST1/2 [34]. In addition, YAP expression was also induced by PI3K/AKT signaling, which was also repressed along with FGFR4 inhibition [35, 36]. However, in the present study, we found that the phosphorylation of MST1/2 and LAST1/2 was only slightly changed after the addition of FGFR4 inhibitor (Fig. S8C), and the phosphorylation and overall levels of YAP were not notably affected (Fig. 5D). This might occur because the compensatory accumulation of EZH2 after Roblitinib treatment may regulate the expression of YAP. It has been reported that EZH2 and DNMT1 can negatively regulate WWC1/2 [37–39], a negative regulator upstream of YAP, thereby activating the expression of YAP. Thus, the stabilization of YAP levels may be the key reason why single inhibition of FGFR4 or EZH2 can inhibit cell proliferation and induce apoptosis, but cannot fully eliminate HCC cells. Therefore, only when FGFR4 and EZH2 signaling were simultaneously suppressed would YAP expression decline dramatically and ultimately synergistically cause massive apoptosis in tumor cells. Our study identified a new regulatory pathway that directly/coordinately regulates YAP signaling to induce HCC apoptosis independently of the canonical Hippo signaling pathway.

## Conclusions

In summary, our study revealed that the co-administration of FGFR4 inhibitor with EZH2 inhibitor could significantly inhibit tumor growth, which may be a potential option for the future clinical treatment of HCC.

## Supplementary Information

**Additional file 1: Fig. S1. **FGFR4 and EZH2 expression are strongly correlated with poor prognosis in HCC patients. **Fig. S2. **FGFR4 inhibitor treatment represses the viability of HCC cells. **Fig. S3. **Elevated EZH2 levels lead to antagonism of HCC against FGFR4 inhibitors. **Fig. S4. **Combination of Roblitinib and CPI-169 synergistically inhibits the HCC cell growth. **Fig. S5. **Toxicity evaluation of the combination treatment of Roblitinib and CPI-169 in zebrafish. **Fig. S6. **Toxicity evaluation of the combination treatment of Roblitinib and CPI-169 in mice. **Fig. S7. **Combination of Roblitinib and CPI-169 synergistically induces HCC cell apoptosis. **Fig. S8. **Combination of Roblitinib and CPI-169 synergistically inhibits the YAP signaling. **Fig. S9. **Overexpression of YAP1^S127A^ antagonizes the synergistic effect of Roblitinib and CPI-169 in HCC cells. 

**Additional file 2: Table S1. **List of the qRT-PCR primers.

**Additional file 3: Table S2. **Information of the antibodies.

## Data Availability

Original RNA-sequencing data are available in the NCBI BioProject database (https://www.ncbi.nlm.nih.gov/bioproject/; BioProject ID: PRJNA906754). The datasets generated and/or analyzed during the current study are available from the corresponding author upon reasonable request.

## Abbreviations

HCC: Hepatocellular carcinoma
FGFR4: Fibroblast growth factor receptor 4
EZH2: Enhancer of zeste homology 2
PRC2: Polycomb repressive complex 2
NFKB2: Nuclear factor kappa B subunit 2
YAP: Yes-associated protein
MST1/2: Mammalian sterile 20-like kinase 1/2
LATS1/2: Large tumor suppressor kinase 1/2
CI: Combination index
GSEA: Gene set enrichment analysis
