# Supplementary material for: FGFR4 and EZH2 inhibitors synergistically induce hepatocellular carcinoma apoptosis via repressing YAP signaling
Source: J Exp Clin Cancer Res. 2023 Apr 22;42:96. doi: 10.1186/s13046-023-02659-4 (PMC10122280; doi:10.1186/s13046-023-02659-4)
Supplement: Supplementary file 1 — Additional file 1: Fig. S1. FGFR4 and EZH2 expression are strongly correlated with poor prognosis in HCC patients. Fig. S2. FGFR4 inhibitor treatment represses the viability of HCC cells. Fig. S3. Elevated EZH2 levels lead to antagonism of HCC against FGFR4 inhibitors. Fig. S4. Combination of Roblitinib and CPI-169 synergistically inhibits the HCC cell growth. Fig. S5. Toxicity evaluation of the combination treatment of Roblitinib and CPI-169 in zebrafish. Fig. S6. Toxicity evaluation of the combination treatment of Roblitinib and CPI-169 in mice. Fig. S7. Combination of Roblitinib and CPI-169 synergistically induces HCC cell apoptosis. Fig. S8. Combination of Roblitinib and CPI-169 synergistically inhibits the YAP signaling. Fig. S9. Overexpression of YAP1S127A antagonizes the synergistic effect of Roblitinib and CPI-169 in HCC cells. [file 13046_2023_2659_MOESM1_ESM.docx]

**Supplementary Figure legends**

**Fig. S1 FGFR4 and EZH2 expression are strongly correlated with poor prognosis in HCC patients.** (A) Relative expression of FGFR4 and EZH2 in HCC tissues compared with in paracancerous liver tissues in The Cancer Genome Atlas (TCGA) dataset. (B) TPM of FGR4 (left) and EZH2 (right) in HCC tissues and paracancerous liver tissues in TCGA dataset. (C-D) High expression levels of FGFR4 (C) and EZH2 (D) were associated with worse disease-free survival, higher individual cancer stages, and higher tumor grade in HCC patients.

**Fig. S2 FGFR4 inhibitor treatment represses the viability of HCC cells.** (A) Cell viability of HepG2, SMMC-7721, MHCC97H, MHCC97L and Huh7 cell lines was evaluated by the CCK-8 following increasing concentrations of Roblitinib treatment for 48 h. Data are presented as mean ± SEM (n = 3). (B-C) Cell viability of HepG2, SMMC-7721 and Huh7 cell lines was evaluated by the CCK-8 following increasing concentrations of BLU9931 (B) and H3B-6527 (C) treatment for 48 h. Data are presented as mean ± SEM (n = 3). (D) Bubble chart showed the KEGG pathway analysis of differentially expressed genes following Roblitinib treatment for 48h. P-values < 0.05 was regarded as statistically significant. (E) Heatmap showed the expression levels of 24 genes related to NF−kappa B signaling pathway determined by RNA-Seq in HepG2 cells following Roblitinib treatment for 48 h.

**Fig. S3 Elevated EZH2 levels lead to antagonism of HCC against FGFR4 inhibitors.** (A-B) Cell viability of SMMC-7721 (A) and Huh7 (B) cell lines transfected with empty vector control (Con), FLAG-EZH2 (EZH2-Ov) and EZH2-siRNAs was evaluated by the CCK-8 following increasing concentrations of Roblitinib treatment for 48 h. Data are presented as mean ± SEM (n = 3). (C) Crystal violet staining of SMMC-7721 cell lines transfected with empty vector control (Con), FLAG-EZH2 (EZH2-Ov) and EZH2-siRNAs following Roblitinib treatment for 48 h. Scale bars: 1 cm. (D) EdU assays of 7721 cells transfected with empty vector control (Con), FLAG-EZH2 (EZH2-Ov) and EZH2-siRNAs following Roblitinib treatment for 48 h (D). Scale bars: 100 μm. (E) Measurement of the cell numbers in (D). Data are presented as mean ± SEM (n = 3, two-way ANOVA with Sidak’s multiple comparison test, ***p < 0.001, ****p < 0.0001, ns, no significance).

**Fig. S4 Combination of Roblitinib and CPI-169 synergistically inhibits the HCC cell growth.** (A-E) Cell viability of HepG2 (A), SMMC-7721 (B), MHCC97H (C), MHCC97L (D) and Huh7 (E) cell lines was evaluated by the CCK-8 following increasing concentrations of CPI-169, Roblitinib or CPI-169 + Roblitinib treatment for 48 h. Data are presented as mean ± SEM (n = 3). (F-J) Drug interaction analysis between CPI-169 and Roblitinib with indicated ratio in HepG2 (F), SMMC-7721 (G), MHCC97H (H), MHCC97L (I) and Huh7 (J) cell lines. The CI values less than 1.0, approximately 1.0, and greater than 1.0 indicate synergism, additive, and antagonism, respectively. (K) Photograph of HepG2 and MHCC97L cell lines following CPI-169, Roblitinib or CPI-169 + Roblitinib treatment for 48 h. Scale bars: 100 μm. (L) Colony formation assay of Huh7 and MHCC97H cell lines following CPI-169, Roblitinib or CPI-169 + Roblitinib treatment for 14 days. Scale bars: 1 cm. Data are presented as mean ± SEM (n = 3, two-way ANOVA with Sidak’s multiple comparison test, *p < 0.05, ****p < 0.0001). (M) Crystal violet staining of HepG2 and SMMC-7721 cell lines transfected with Negative control (NC), EZH2-siRNA, FGFR4-siRNA and EZH2-siRNA + FGFR4-siRNA for 48 h. Scale bars: 1cm.

**Fig. S5 Toxicity evaluation of the combination treatment of Roblitinib and CPI-169 in zebrafish.** (A-D) Toxicity evaluation of CPI-169 in zebrafish. Shown were the photograph of zebrafish treated with CPI-169 for 12 h, 24 h, 48 h and 72 h (A) and their corresponding survival rates (B), body length (C) and heart beats/min (D). (E-H) Toxicity evaluation of the Roblitinib in Zebrafish. Shown were the photograph of zebrafish treated with Roblitinib for 12 h, 24 h, 48 h and 72 h (E) and their corresponding survival rates (F), body length (G) and heart beats/min (H). (I-L) Toxicity evaluation of the combination of Roblitinib + CPI-169 in Zebrafish. Shown were the photograph of zebrafish treated with Roblitinib + CPI-169 for 12 h, 24 h, 48 h and 72 h (I) and their corresponding survival rates (J), body length (K) and heart beats/min (L). Scale bars: 500 μm. Data are presented as mean ± SEM (n = 10, one-way ANOVA with Tukey’s multiple comparison test, ns, no significance).

**Fig. S6 Toxicity evaluation of the combination treatment of Roblitinib and CPI-169 in mice.** (A) Body weight of the mice under treatment. Mice with SMMC-7721 xenografts were treated with vehicle (n = 6), CPI-169 (n = 6), Roblitinib (n = 6), or in combination (CPI-169 + Roblitinib; n = 6) for 2 weeks. Values are represented as mean ± SEM; Two-way ANOVA with Sidak’s multiple comparison test, ns, no significance. (B) Hematoxylin and eosin staining of the heart, liver, spleen, lung and kidney sections of the representative mice bearing SMMC-7721 xenografts treated with CPI-169 and/or Roblitinib for 2 weeks. Scale bars: 300 μm.

**Fig. S7 Combination of Roblitinib and CPI-169 synergistically induces HCC cell apoptosis.** (A) Photograph of HepG2 and SMMC-7721 cell lines following CPI-169, Roblitinib or CPI-169 + Roblitinib treatment for 48 h. Scale bars: 100 μm. (B) Flow cytometric analysis of apoptosis in HepG2 cells following CPI-169, Roblitinib or CPI-169 + Roblitinib treatment for 48 h. (C) Western blot analysis of the indicated protein levels in HepG2 cells following CPI-169, Roblitinib or CPI-169 + Roblitinib treatment for 48 h. Data are presented as mean ± SEM (n = 3, two-way ANOVA with Sidak’s multiple comparison test, *p < 0.05, **p < 0.01, ***p < 0.001, ****p < 0.0001, ns, no significance).

**Fig. S8 Combination of Roblitinib and CPI-169 synergistically inhibits the YAP signaling.** (A) Heatmaps showed the expression level of genes determined by RNA-Seq in HepG2 cells following CPI-169, Roblitinib or CPI-169 + Roblitinib treatment for 48 h. (B) Numbers of the differentially expressed genes between the treatment group and the control group in (A). (C) Western bolt detected the expression level of MST1/2, p-MST1/2 (top) and LATS1/2 and p-LATS1/2 (bottom) in HepG2 cells after CPI-169, Roblitinib or CPI-169 + Roblitinib treatment for 48 h. (D) qPCR analysis of YAP1 in HepG2 cells following the treatment in (A). Data are presented as mean ± SEM (n = 3, one-way ANOVA with Tukey’s multiple comparison test, ***p < 0.001, ****p < 0.0001). (E) Venn diagram displayed overlapping genes from 2 different YAP1 ChIP-seq datasets to identify 741 accordant YAP1 target genes. The datasets included the human cholangiocarcinoma cell lines CClp1 (GSM1524329, left) and HuCCT-1 (GSM1667157, right). (F) RNA-Seq analysis of the mean expression of the 141 YAP1 target genes under different treatment conditions. The abscissa represented the indicated treatment conditions, while the ordinate represented log 10 FPKM. Data are presented as mean ± SEM (n = 3, one-way ANOVA with Tukey’s multiple comparison test, ****p < 0.0001, ns, no significance).

**Fig. S9 Overexpression of YAP1^S127A^ antagonizes the synergistic effect of Roblitinib and CPI-169 in HCC cells.** (A-B) Cell viability of the HepG2 and HepG2 YAP1^S127A^ (A), SMMC-7721 and SMMC-7721 YAP1^S127A^ (B) cell lines was evaluated by the CCK-8 following increasing concentrations of CPI-169 or Roblitinib treatment for 48 h. Data are presented as mean ± SEM (n = 3). (C) Colony formation assay of SMMC-7721 and SMMC-7721 YAP1^S127A^ cell lines following CPI-169, Roblitinib or CPI-169 + Roblitinib treatment for 14 days. Scale bars: 1cm. (D) Luciferase assay for YAP/TAZ activity in HepG2, HepG2 YAP1^S127A^, SMMC-7721 and SMMC7721 YAP1^S127A^ cell lines following CPI-169 + Roblitinib treatment for 48 h. Data are presented as mean ± SEM (n = 3, two-way ANOVA with Sidak’s multiple comparison test, *p < 0.05, **p < 0.01, ***p < 0.001, ****p < 0.0001, ns, no significance). (E) qPCR analysis of the representative YAP target genes from typical pathway related to cell proliferation, cell migration and anti-apoptosis in HepG2 and HepG2 YAP1^S127A^ cell lines following the CPI-169 + Roblitinib treatment for 48 h. Data are presented as mean ± SEM (n = 3, two-way ANOVA with Sidak’s multiple comparison test, *p < 0.05, **p < 0.01, ***p < 0.001, ****p < 0.0001, ns, no significance).

**Supplementary Table S1** List of the qRT-PCR primers.

**Supplementary Table S2** Information of the antibodies.
